# The Role of Dietary Carbohydrates in Gestational Diabetes

**DOI:** 10.3390/nu12020385

**Published:** 2020-01-31

**Authors:** Vikkie A. Mustad, Dieu T.T. Huynh, José M. López-Pedrosa, Cristina Campoy, Ricardo Rueda

**Affiliations:** 1 R&D Department, Abbott Nutrition, Columbus, OH 43219, USA; vikkie.mustad@abbott.com; 2 R&D Department, Abbott Nutrition, Singapore 138668, Singapore; dieu.huynh@abbott.com; 3 R&D Department, Abbott Nutrition, 18004 Granada, Spain; jose.m.lopez@abbott.com; 4 Department of Paediatrics, University of Granada, 18071 Granada, Spain; ccampoy@ugr.es; 5 EURISTIKOS Excellence Centre for Paediatric Research, University of Granada, 18071 Granada, Spain

**Keywords:** gestational diabetes mellitus, pregnancy, dietary carbohydrates, diabetes-specific formula, continuous glucose monitoring

## Abstract

Gestational diabetes (GDM) is hyperglycemia that is recognized for the first time during pregnancy. GDM is associated with a wide range of short- and long-term adverse health consequences for both mother and offspring. It is a complex disease with a multifactorial etiology, with disturbances in glucose, lipid, inflammation and gut microbiota. Consequently, its management is complex, requiring patients to self-manage their diet, lifestyle and self-care behaviors in combination with use of insulin. In addition to nutritional recommendations for all pregnant women, special attention to dietary carbohydrate (CHO) amount and type on glucose levels is especially important in GDM. Dietary CHO are diverse, ranging from simple sugars to longer-chain oligo- and poly- saccharides which have diverse effects on blood glucose, microbial fermentation and bowel function. Studies have established that dietary CHO amount and type can impact maternal glucose and nutritional recommendations advise women with GDM to limit total intake or choose complex and low glycemic CHO. However, robust maternal and infant benefits are not consistently shown. Novel approaches which help women with GDM adhere to dietary recommendations such as diabetes-specific meal replacements (which provide a defined and complete nutritional composition with slowly-digested CHO) and continuous glucose monitors (which provide unlimited monitoring of maternal glycemic fluctuations) have shown benefits on both maternal and neonatal outcomes. Continued research is needed to understand and develop tools to facilitate patient adherence to treatment goals, individualize interventions and improve outcomes.

## 1. Introduction

Gestational diabetes (GDM) is one of the most common adverse medical conditions of pregnancy, and its prevalence is rising as part of the global diabetes pandemic, both in developed and developing countries. GDM is complex and multifactorial, with several aspects contributing to explain its pathophysiology, although this is not fully clarified yet.

Nutrition in general and some particular nutritional compounds, such as carbohydrates (CHO) and fiber, contribute to regulate glycemic index and glycemic response, and consequently can influence establishment and evolution of gestational diabetes during pregnancy as well as risk of clinical outcomes both in the mother and the infant. Consequently, nutrition may be a key tool for prevention and management of gestational diabetes.

After a brief overview of the prevalence, short- and long-term health consequences and pathophysiology of GDM, this review will focus on what is known about the role of dietary carbohydrates in its prevention and treatment. Nutrition recommendations from professional health groups will be summarized, with a focus on the specific research on dietary carbohydrate quantity and quality that informs the basis for carbohydrate recommendations. Finally, the use of novel approaches such as specialized nutritional supplements and continuous glucose monitoring to help patients adhere to treatment goals will be discussed.

## 2. Background

### 2.1. Prevalence Worldwide and the Trends over the Past Decade

Gestational diabetes (GDM) is hyperglycemia that is recognized for the first time during pregnancy. It encompasses undiagnosed type 2 diabetes and hyperglycemia which develops later in pregnancy [1]. It is one of the most common adverse medical conditions of pregnancy, and its prevalence is rising as part of the global diabetes pandemic. It is estimated that GDM affects around 21.3 million or one in six live births in 2017 [2]. Studies have shown that GDM increases with a faster rate in lower- and middle-income countries than high-income countries, especially over the past decade [1,2,3]. Yan et al. reported the GDM prevalence increased from 15.5% to 19.9% from 2012 to 2017 in Xiamen, China [1]. Lavery et al. reported about 5.5% increase in GDM prevalence in the USA over a 20-year period from 1990 to 2010 [3]. A study in Spain examining the trend of GDM prevalence showed an increase of 4.8% over a period of 9 years from 2006 to 2015 [4]. According to the International Diabetes Federation, South East Asia had the highest prevalence of GDM with 26.6% followed by Middle East/North Africa (18%), Europe (14%) and Africa (9.5%) [2]. Because of significant health and economic burdens, the increasing prevalence of GDM in most populations has become a global health challenge, especially in low- and middle-income countries.

### 2.2. Short- and Long-Term Health Consequences for Mothers and Offspring

In the short-term, GDM increases pregnancy and birth complications. Women with GDM are more likely to develop gestational hypertension and preeclampsia. A population-based retrospective cohort including 426,296 deliveries over a 10-year period reported a 90% higher risk of preeclampsia in women with GDM than those without GDM [5]. Pre-pregnancy obesity, excessive gestational weight gain and poor glycemic control are linked with greater risk of gestational hypertension and pre-eclampsia in women with GDM [6]. GDM is considered an independent risk factor for newborn large for gestational age (LGA) and macrosomia. A meta-analysis reported the pooled odds ratio of macrosomia was 5.5 folds higher in women with GDM than those without GDM [7]. Additionally, the risk for LGA increases as fasting and two-hour post-oral glucose tolerance test (OGTT) glucose increase during pregnancy. Farrar et al. studied the association between blood glucose and perinatal outcomes in a meta-analysis involving up to 207,172 women [8]. They found that the odds ratios for LGA per 18 mg/dL increase in fasting and two-hour post-OGTT glucose concentrations were 2.15 (95% confidence interval 1.60 to 2.91) and 1.20 (1.13 to 1.28), respectively. Infants born LGA and with macrosomia increased instrumental delivery, caesarean section, premature birth and shoulder dystocia [8,9]. Such pregnancy and neonatal complications were associated with delayed initiation of breastfeeding, lower rates of breastfeeding and breastfeeding duration in women with GDM [10,11]. Because breastfeeding has been shown to be a protective factor for the development of T2DM in GDM mothers and in their offspring [12,13], suboptimal breastfeeding has an implication in future health risk for both mother and child.

Additionally, these pregnancies and neonatal complications impose substantial economic burdens. A burden-of-illness study on GDM conducted in China has shown that the cost of a pregnancy with GDM was 95% more than for a pregnancy without GDM, due to additional expenses for GDM diagnosis and management, and for mother and neonatal complications. With a number around 2.9 million pregnancies affected by GDM in 2015, the estimated annual economic burden of GDM was an international $5.59 billion from incremental direct medical costs [14].

In the long-term, GDM increases the risk—in both mother and offspring—of type 2 diabetes, metabolic syndrome and obesity. In their systematic review, Hopmans et al. [15] showed that the increased risk of type 2 diabetes and cardiovascular disease was 13 times and two times higher, respectively, in women with GDM when compared with healthy control [15]. In a recent meta-analysis, Kramer et al. investigated the association between GDM and long-term cardiovascular disease in 5,390,591 women. They found that GDM was associated with a 2.3-fold increased risk of cardiovascular events in the first decade postpartum when compared with women without GDM [16].

In children, intrauterine exposure to GDM increases the risk for overweight/obesity and abnormal glucose tolerance. A total of 26,509 children born to mothers with and without GDM were included in a meta-analysis to investigate the association of maternal hyperglycemia exposure during pregnancy with obesity and abnormal glucose tolerance in offspring [17]. The rate of obesity or overweight in children aged 2–17 years was 1.35 times higher in the offspring of GDM mothers. This increased risk was highest in the age group of 11 years and above [17]. Additionally, the children of women with GDM had higher two-hour plasma glucose through early adulthood (pooled MD: 0.43 mmol/L, 95% CI: 0.18–0.69), [17]. Another study reported a four-fold increased risk for metabolic syndrome in children from GDM mothers, with the risk increasing significantly with increasing maternal fasting and two-hour blood glucose [18]. Overwhelmingly, GDM has significant and adverse implications on the future burden of non-communicable diseases in both mother and offspring.

## 3. Key Aspects of GDM Pathophysiology

GDM is a complex disease with a multifactorial etiology; however, the exact mechanisms involved in its pathophysiology are not fully clarified [19]. The purpose of this section is to review the key aspects implicated in its onset and recent advances regarding GDM.

### 3.1. Glucose and Lipid Metabolism 

During normal pregnancy, the body undergoes numerous metabolic and immunological changes that are designed to maintain maternal and fetal health. One of the major metabolic changes is related to glucose metabolism [20]. Glucose metabolism changes according to the course of pregnancy and fetal needs. At first, fasting glucose levels drop, likely due to both increased maternal blood volume and the fetal use. To adjust to this physiological change, pancreatic β cells suffer hyperplasia and hypertrophy to increase insulin secretion, maintaining maternal euglycemic state and preserving the embryo [19]. Another common change that occurs during normal pregnancy is a reduction of insulin sensitivity and an increase of insulin resistance. This is normally seen in normal pregnancies, presumably to spare the glucose for the fetus, and can be attributed to the production of placental hormones such as estrogens, progesterone, placental lactogen, placental growth hormone, leptin and cortisol and may be intensified by genetic susceptibility and/or mother’s lifestyle [21]. 

Exacerbated peripheral insulin resistance during pregnancy and poor β-cell adaptation likely contribute to GDM. Decreased insulin-stimulated glucose disposal by 22% has been observed in GDM when compared with normal pregnancy [22]. As compared to normal pregnancy, impaired skeletal muscle glucose uptake in women with GDM is usually explained by the failure of insulin signaling, due to an additional decrease in tyrosine phosphorylation of the insulin receptor, which inhibits insulin signaling from activating GLUT4 translocation. Decreased insulin-stimulated glucose uptake can trigger failure of the β-cell and hyperglycemia in GDM [23]. Indeed, β-cell failure or insufficiency were described by Buchanan et al. [24], showing that insulin secretion increases throughout pregnancy in both women with and without GDM, but beginning at a lower starting point in women with GDM.

Along with changes in insulin resistance and the subsequent response of pancreatic β-cells, hepatic glucose production is also significantly altered in women with GDM. Basal endogenous glucose production increases in a similar way in women with and without GDM [22]. However, in late gestation the ability of insulin to suppress gluconeogenesis was lower in GDM as compared to the normal pregnant women.

Major changes in lipid metabolism also occur during pregnancy [25]. In the mother, during the early stage of pregnancy there is an increase in the fat depot accumulation as result of increased lipid synthesis and hyperphagia. During this period, these lipidic disturbances are caused by enhanced adipose insulin responsiveness that facilitates the accumulation of circulating lipids in the adipose tissue. However, in late gestation, and as consequence of the insulin resistant condition, adipose lipolytic activity is increased with a parallel reduction in the lipoprotein lipase activity and fatty acid synthesis in the adipose tissue. These changes determine the development of maternal hypertriglyceridemia in normal pregnancy [26]. In GDM, the pronounced peripheral resistance and inadequate β-cell function cause these alterations to be exacerbated, resulting in enhanced lipolysis and ketogenesis. Under these circumstances, the dyslipidemia in diabetic pregnancy compared with normal pregnancy actively contributes to the chronic maternal insulin resistance and increases the exposure of lipids to the fetus, contributing to fat accumulation, macrosomia and LGA. Enhanced levels of small size and dense LDL particles have been consistently found in GDM [27] regardless the circulating concentrations of LDL-cholesterol [28].

### 3.2. Inflammation 

A tightly-regulated balance between pro- and anti-inflammatory cytokines is believed to be necessary for normal implantation, trophoblast invasion and placentation [29]. In early and late-pregnancy, elevated secretion of pro-inflammatory cytokines (TNF- α and Interleukin (IL)-6) and reduced levels of anti-inflammatory cytokines (Il-10 and IL-4) are observed in women developing GDM [30], suggesting that inflammation may be involved in the development of insulin resistance associated with GDM. Pro-inflammatory cytokines can alter the insulin signaling pathway by dampening insulin receptor (IR) tyrosine kinase activity, amplifying serine phosphorylation of IRS-1, or by altering the STAT3-SOCS3 pathway. In a study by Kirwan et al., 2002 [31] and corroborated by Catalano and coworkers, 2014 [22] they showed that plasma TNF α was the most strongly correlated factor with insulin sensitivity. Similarly, placental gene expression of TNF-α, IL-1β and their receptors have been reported to be increased in GDM. Besides that, in a study published in October 2019 [32], another pro-inflammatory cytokine (IL-34) which operates as a ligand for colony-stimulating factor-1 receptor (CSF-1R) was discovered to contribute to the apoptosis of pancreatic β cells, playing a crucial role in the development of GDM [32].

### 3.3. Gut Microbiota 

The normal gut microbiota composition appears to undergo important shifts during normal pregnancy. In late pregnancy, the gut microbiota pattern resembles the disruptive gut microbiota composition to that of adults with type 2 diabetes [33]. Individuals with insulin resistance show significant changes in the *Firmicutes/Bacteroidetes* ratio, with a reduction in butyrate-producing bacteria compared to healthy individuals [34]. Fugmann et al. (2015) [35] analyzed stool microbiota both in insulin resistant women with a recent history of GDM and women after a normoglycemic pregnancy. They found that women with a *Prevotellaceae*-dominated intestinal microbiome were overrepresented in the GDM group. Similarly, Bassols et al. (2012) [36] extracted and analyzed microbial DNA and RNA from the appendix contents of eight insulin resistant and eight insulin-sensitive obese subjects. They concluded that gut RNA microbial profile varies in accordance with insulin action. Recently, Crusell et al. (2018) [33] showed that gut microbiota of women with GDM are distinct from microbiota changes of normal pregnant women, including phylum and genus levels. In this study, there was a difference in the abundance of 17 species level operational taxonomic units (OTUs) in GDM compared with the normoglycemic pregnant women. Although different studies report the link between the gut microbial profile and insulin resistance, the potential pathophysiological role of gut bacteria in GDM remains still unknown.

## 4. Role of Dietary Carbohydrates in GDM

Dietary carbohydrates (CHO) are an important energy source for both mother and fetus, and all pregnant women need at least 175 g of CHO, including 28 g of fiber per day [37]. However, for women with GDM, there is an additional need to pay careful attention to amount and type of CHO as it is well-established that dietary CHO have the greatest impact on blood glucose. Dietary CHO are diverse in structure with variable effects on blood glucose responses and other physiological outcomes [38]. Many dietary CHO (glucose, sucrose, cooked starches found in pastas, potatoes, white bread) are readily digested and absorbed in the small intestines, and these contribute to a rapid increase in blood glucose. Some dietary CHO have a structure that makes them resistant to digestion—or completely non-digested—and these result in a small and/or slower increase in blood glucose (e.g., low glycemic index, LGI). Components of CHO that are not digested (fiber) pass through the small and large intestine where they can provide important physiological benefits such as stimulate incretin production, serve as an energy source for colonic microbiota, and promote normal bowel movements.

Early studies established that elevated postprandial glucose responses contribute to an increased glucose transport to the fetus and hyperglycemia correlates significantly with infant size and/or adiposity [39,40]. Studies also have established that maternal glucose responses can be markedly influenced by the total amount of CHO [39,41,42] or the type of CHO [43] consumed. Evidence from preclinical studies [44,45] using animals models of gestational diabetes shows that negative effects of feeding high-GI sucrose and maltodextrin on the pathophysiology of GDM in dams and their offspring can be reversed by substituting these rapidly-metabolized CHO with more slowly-digesting CHO (i.e., isomaltulose and resistant maltodextrins). As a result, in order to improve maternal fasting and postprandial glucose during GDM, current dietary guidelines recommend either to restrict CHO intake or to replace high glycemic/rapidly-digesting (HGI) CHO with those that are more slowly digesting (Table 1).

However, as commented in numerous publications [46,47,48,49], these dietary recommendations are limited by the lack of robust evidence. Randomized nutritional intervention trials that have evaluated dietary strategies focusing on CHO in women with [50,51,52,53,54,55,56,57,58] or at risk of [59,60,61,62] GDM have not shown consistent benefits on maternal or infant outcomes. The details of the studies, their dietary interventions and outcomes have been critically reviewed in other recent publications [46,47,48,49]. Overall, it is widely viewed that these studies suffer from small sample size and thus are underpowered and observed benefits on LGA or other infant and maternal outcomes are not consistent across studies. The most current Cochrane analysis [63] did not show significant maternal and/or infant benefits; in contrast, Yamamoto et al. [64] focused their primary analysis on maternal glycemic outcomes and concluded that interventions that improved maternal glycemic control would improve infant birthweight outcomes. In agreement was another meta-analysis by Wan et al. [65] who focused their meta-analysis on dietary intervention strategies to include all Chinese-language studies. Their conclusion was that CHO-modified diets were associated with improved glycemic control and infant birth outcomes in ethnic Chinese women with GDM.

Studies have evaluated nutritional interventions for preventing GDM [59,60,61,62,66] Most have combined increasing physical activity with energy restriction through reducing on the quantity and/or changing the type of CHO to reduce or slow weight gain during early pregnancy. Studies and their findings are diverse. In the LIMIT trial [59] over 2000 pregnant overweight and obese pregnant women were randomized before 20 weeks of gestation to standard of care or an intensive lifestyle arm including advice to reduce intake of refined CHO. Babies born to women in the intensive lifestyle intervention were significantly less likely to be LGA, have respiratory distress syndrome and had shorter hospital stays. In the GI Baby 3 study of 139 women at high risk of GDM [60], those following low-GI diet advice required less insulin to maintain normoglycemia (*p* = 0.007) compared to a group following a high-GI diet. Walsh et al. [61], in the ROLO study, evaluated 800 women in their second half of pregnancy who were at high risk for GDM having previously delivered an infant weighting greater than 4 kg. Pregnant women who were randomized to receive low-GI diet advice had significantly lower gestational weight gain and less maternal glucose intolerance compared to those following standard of care. However, the incidence of LGA infants was not reduced. The UPBEAT study [0] randomized 1555 obese women to receive a standard of care or an intensive behavioral intervention to increase physical activity and improve diet quality with an emphasis on low-GI foods. Despite improvements in gestational weight gain, the intervention was not associated with additional benefits.

Most recently, Zhang et al., [67] reviewed the effects of low-GI diets in all pregnant women, both those having healthy pregnancies, at risk for GDM, and those with GDM. In a total of 11 trials involving 1985 women, low-GI diets significantly reduced fasting and two-hour postprandial glucose level. Pregnant women following low-GI diet advice had a higher risk of delivery of low for gestational age neonates; however, there were no significant benefits on maternal or newborn outcomes.

Taken together, a low-GI diet during early pregnancy can improve postprandial glucose and weight gain, at least in some studies. However, these interventions are not adequate to prevent GDM or to consistently reduce the incidence of LGA infants. Whether the type of CHO (low-GI or slowly digesting and low-GI) or additional factors are necessary to offset the rapidly changing and complex pathophysiology that occurs during pregnancy are unknown. Additional insight into the role of CHO can be gained from observational studies that have investigated nutritional intakes and/or patterns before or during pregnancy and GDM; these studies [68,69] show GDM is higher in those having higher intakes of meat and a lower intake of whole grain carbohydrates, fruits, vegetables, and fish. These studies suggest that CHO along with other dietary components are likely involved. A high intake of saturated fat can interfere with insulin signaling, and they can also induce inflammation and endothelial dysfunction, both pathogenic factors in GDM. Amino acids can act as substrates for hepatic glucose production and in hepatic lipotoxicity. Although CHO such as fructose found in fruit are lower GI, more slowly digesting CHO such as those found in whole grains can slow sugar absorption, reducing the demand on cells and insulin signaling mediators. In addition, proper intake of micronutrients and polyunsaturated fats, including those derived from fish and seafood, have anti-inflammatory properties consistently associated with a reduced risk of GDM. Another key consideration in all intervention studies is the ability of the women to adopt the intervention. In their systematic review, Lammimpaa and coauthors concluded that variability in the delivery of dietary interventions is a key factor affecting study outcomes in GDM. Thus, additional research on the benefits of dietary CHO, with or without other nutritional factors, should also pay attention to the consistency of its delivery [70].

## 5. Promising Approaches to Help Patients with GDM Adhere to Dietary Recommendations

GDM is a complex disease requiring patients to self-manage their diet, lifestyle, and self-care behaviors in combination with use of insulin or, in some cases, oral medications such as metformin or glyburide [71]. Trying to engage pregnant women with (or at risk of) GDM to change their diet is especially challenging; although most women are especially attuned to the health of their developing fetus, food cravings, palatability, hunger and time pressure are cited as barriers to adherence to prescribed diets [72].

Meal replacements can be helpful for people not only for being convenient, but also for providing known calorie amounts with specific macro- and micro-nutrient levels that facilitate meal planning.

When used as part of lifestyle intervention in diabetes prevention programs, meal replacements have been shown to promote weight loss and reduction in the incidence of type 2 diabetes in overweight/obese people at increased risk of developing type 2 diabetes [73,74]. There is good evidence that meal replacement as part of a lifestyle intervention is effective in promoting weight loss and improving metabolic outcomes in individuals with type 2 diabetes [75,76] The current diabetes guidelines from the American Diabetes Association [77], the Canadian Diabetes Association [78], and Diabetes UK [79] have recommended the use of meal replacements in the management of individuals with diabetes. 

The role of meal replacements in preventing the development of GDM has not been well studied. Given the risk of pregnancy complications such as gestational diabetes, macrosomia and Cesarean delivery, and the risk of adverse metabolic health outcomes associated with excessive gestational weight gain (GWG), recently Phelan et al. [80] conducted an RCT involving 257 overweight and obese pregnant women (mean ± SD: 13.6 ± 1.8 weeks of gestation) to evaluate the effect of a behavioral lifestyle intervention (*n* = 129) with partial meal replacement in comparison of enhanced usual care (*n* = 128) on weekly gestational weight gain (GWG) rate, cardiovascular disease risk factors, and incidence of pregnancy complications. Participants in the enhanced usual care group received all aspects of usual care offered by their prenatal care providers. In addition, they were given the general information about healthy eating, physical activity, and the Institute of Medicine (IOM) recommendations for total gestational weight gain at the time of study randomization. The intervention group received all aspects of enhanced usual care plus a behavioral lifestyle intervention designed to prevent excessive weight gain during pregnancy. Each participant received a 20-min individual, face-to-face counseling session with a study interventionist every two weeks until 20 weeks of gestation and then monthly visits until delivery. To promote weight control, they were instructed to replace two meals with the provided meal replacement shakes or bars and to consume ≥ 1 meal of regular foods and 2–4 healthy snacks/day. The meal replacement products included organic and lactose-free drinks and bars in which 80% of the study’s meal replacements purchased were for organic meal replacement products high in protein, 15% were for the bar also high in protein; and 4% were for powder meal replacement including a standard oral nutritional supplement (4%) and a diabetes-specific formula (1%). The meal plan using partial meal replacement provided a calorie intake of ~18 kcal/kg body weight at study entry in which 30%, 15%–20% and 50–55% of calories were from fat, protein, and carbohydrates, respectively. The intervention group had significantly lower weekly GWG rates compared with the control group (0.33 vs 0.39 kg/week, respectively, *p* = 0.02). In addition, 43% women in the intervention group were less likely to exceed the (IOM) recommendations for total GWG than were those in the control group (OR: 0.57; 95% CI: 0.34, 0.95; *P* = 0.03). From study entry to 35–36 weeks of gestation, when compared with the enhanced usual care, the intervention significantly reduced triglycerides (*P* = 0.03) and resulted in trend reductions in fasting glucose (*P* = 0.09) and systolic blood pressure (*P* = 0.06). Regardless of group assignment, greater increases in GWG rate were associated with increases in insulin (2.63 μU/mL; 95% CI: 1.63, 4.23 μU/mL; *P* < 0.0001), HOMA-IR (2.84; 95% CI: 1.67, 4.85; *P* < 0.0001), and C-peptide (1.52 ng/mL; 95% CI: 1.15, 2.01 ng/mL; *P* < 0.0001). There were no significant differences between groups in triglycerides, HDL cholesterol, LDL cholesterol, total cholesterol, diastolic blood pressure and systolic blood pressure. The incidence of pregnancy complications was similar between groups. The study has suggested that partial meal replacements as part of a behavioral lifestyle intervention is effective for GWG control in women who are overweight and obese. There is a need for further research on its effectiveness before it could be considered as part of the usual prenatal care for pregnant women with obesity.

### 5.1. Evidence for the Use of Complete and Balanced Nutritional Supplements Containing Low-Glycemic and Slowly Digested Carbohydrates on GDM Outcomes

Specialized diabetes-specific formulas (DSF) are designed using low glycemic index and slowly digested carbohydrates and monosaturated fatty acids to support glycemic control [81,82,83]. Systematic reviews and meta-analyses have shown that DSF as part of lifestyle interventions effectively improves glycemic control and reduces cardiometabolic risks [84,85]. The effects of DSF on blood glucose management in women with GDM were investigated in a few studies. Yu et al. [86] conducted a randomized, controlled, unblinded study to investigate the effects of a DSF on postprandial blood glucose and pregnancy outcomes in Chinese women with GDM from October 2011 to January 2012. A total of 69 women diagnosed with GDM around 26 weeks of gestation were randomized to the intervention consisting of individualized dietary recommendations using DSF (*n* = 32) or receiving individualized dietary recommendation only (*n* = 31), as the control group. The DSF was consumed twice daily to replace regular milk during breakfast and a meal. The 2-h postprandial blood glucose was measured weekly over a period of eight weeks from enrollment to the week prior to delivery. When compared with the control group, the intervention group had significantly lower 2-h postprandial blood glucose levels based on the General Linear Model Repeated Measures (*P* < 0.01) and significantly lower HbA1c (5.5% vs. 5.7%; *P* < 0.05). Pregnancy outcomes were found to be in favour of the intervention group in which the intervention had significantly lower incidences of premature rupture of membranes (9.7% vs, 34.5%; *P* < 0.05), polyhydramnios (12.9% vs. 37.9%; *P* < 0.05), and neonatal pneumonia (6.5% vs. 31.0%; *P* < 0.05), and significantly lower birth weight (3346 g vs. 3549 g; *P* < 0.05) [86].

Liu et al. [87] evaluated the effects of a DSF on fasting blood glucose, 2-h postprandial blood glucose, triglycerides and total cholesterol in a 2-week randomized, controlled trial on 40 GDM pregnant women. All participants received individualized dietary counseling in which the total calories were calculated to achieve an ideal body weight with 30–38 kcal/kg body weight. They were instructed to consume six meals a day including three normal meals plus three extra meals to achieve the target calories. For women in the intervention group (*n* = 20), a DSF was given in two extra meals at 9:00 am and 3:00 pm. The calories from two servings of a DSF account for about 25% of total daily calories. Women in the control group (*n* = 10) were instructed to select CHO such as skim milk, fruits and oatmeal as part of a diabetes meal plan. There were no significant differences in 2-h postprandial blood glucose (at 11 am) between the DSF (8.1 ± 0.9 mmol/L) and the control groups (8.4 ± 1.1 mmol/L) at baseline. Both study groups showed a significant reduction in 2-h postprandial glucose levels (at 11 am) from baseline after two weeks of intervention. However, the intervention group achieved a significantly greater improvement compared with the control (6.6 ± 1.5 mmol/L and 7.1 ± 1.3 mmol/L, respectively, between-group *P* < 0.05). Similar findings were observed for 2-h postprandial blood glucose for the extra meal in the afternoon (at 5 pm). No differences in serum triglyceride and total cholesterol concentrations were found after two weeks of intervention [87].

The effects of a DSF on blood glucose control were evaluated in another randomized, controlled trial with nine weeks of intervention involving 70 Chinese GDM pregnant women [88]. Both the intervention (*n* = 34) and the control (*n* = 36) groups received a diabetes diet in which the total daily calories were distributed as follows: 10%, 30%, 30% and 30% for breakfast, lunch, dinner and snacks, respectively. For the intervention group, a DSF was used as a snack replacement. Fasting plasma glucose levels and 2-h postprandial blood glucose levels were measured on a weekly basis over the period of nine weeks. There were some differences in fasting plasma glucose levels and 2-h postprandial blood glucose levels between the groups over a 9-week period. However, it is unclear if the intervention group had better glycemic control because the results of using a statistical test to compare the differences was not mentioned [88].

Overall, there is evidence that use of a DSF as a partial meal or snack replacement in a diabetes meal plan resulted in a greater improvement in glycemic control compared with dietary counseling alone in women with GDM, suggesting it could be an effective treatment for glycemic control in GDM. There are also preliminary findings suggesting its benefits in reducing pregnancy and birth complications. Further studies are needed to confirm this.

### 5.2. Application of Continuous Glucose Monitoring to Facilitate Close Monitoring of Diet on Glucose Control

A treatment goal for GDM to reduce maternal and perinatal outcomes is to maintain a fasting glucose concentration of less than 90–95 mg/dL, one hour postprandial glucose level < 140 mg/dL or 2-h postprandial glucose level of < 120 mg/dL) [89]. The most popular method for daily blood glucose control is self-monitoring blood glucose (SMBG), usually by fingerstick blood sampling at defined times. Use of SMBG readings provide a limited number of measurements and insufficient information of the blood sugar level changes. Recent technology, such as continuous glucose monitoring (CGM), is a promising method for improving the glucose profile by giving a more complete view and thus improving patient treatment and quality of life [90].

CGM comprises a subcutaneous glucose-sensing device and an electrode impregnated with glucose oxidase that allows interstitial glucose levels to be measured approximately every ten seconds and an average value is stored in the monitor every five minutes [90,91]. CGM is an effective tool in the management of pregnant women with type 1 diabetes and has been shown to improve neonatal outcomes. Feig et al., 2017 [92], in the multicenter, open-label, randomized controlled trial LANCET study, examined the effectiveness CGM on maternal glucose control and obstetric and neonatal health outcomes (*n* = 325 women). The authors concluded that the use of CGM during pregnancy is associated with improved neonatal outcomes, which are likely to be attributed to reduced exposure to maternal hyperglycemia.

Additional benefits of CGM include the ability to improve screening protocol [90]. Importantly, the ability to understand normoglycemia and its patterns of variation in nondiabetic pregnant women across the day can allow for future individualized therapies in these patients [93]. Yu et al., 2014 [94] in a prospective cohort study evaluated the effectiveness of CGM on maternal glycemic control and pregnancy outcome in 340 patients with GDM. Patients were allocated in two groups, 190 receiving a routine care self-monitoring of blood glucose (SMBG) and other 150 following the CGM. They concluded that the use of supplementary CGM can improve the glycemic variability and pregnancy outcomes in patients with GDM.

In the last decade, some studies used the CGM technology to facilitate close monitoring of diet on glucose control. Hernandez et al., 2014 [53], in a randomized crossover study, compared two different types of diets (one higher in complex CHO/lower fat and other one lower-CHO/higher fat) using CGM for 72 h to obtain glucose profiles in 16 GDM women. These data show that a diet high in complex CHO and reduced fat still achieved glycemia below current treatment targets and lower postprandial free fat acids. Subsequently, Carreiro et al., 2016 [95] used CGM to more accurately assess the impact of dietary recommendations on women suffering from gestational diabetes and they concluded that dietary counseling was able to keep glucose levels close to those of healthy patients. In a prospective observational study with GDM patients two methods to evaluate glucose readings, CGM and self-monitor blood sugar glucose (SMBG), during Ramadan fasting were compared [96]. They concluded that CGM was effective to detect more hypoglycemia than SMBG in GDM patients.

Although limited, studies have shown that CGM systems have the potential to help the patient and their health care providers manage their day-to-day diet, lifestyle, self-care and medication decisions to achieve treatment goals. However, more research is needed to understand its full potential on both mother and infant outcomes.

## 6. Conclusions

Nutrition is critical to the prevention and treatment of GDM for the health and well-being of both mother and offspring. Close attention to the amount and type of dietary CHO can have important benefits on GDM pathophysiology, but interventions as currently implemented may not be adequate to prevent or treat this complex disease. Continued research is needed to develop tools to facilitate patient adherence to treatment goals, individualize interventions and improve the results.

## Figures and Tables

**Table 1:** Nutrition Recommendations for gestational diabetes mellitus (GDM).

Organization	General Recommendation for GDM	Carbohydrate-Specific Recommendations	Reference/Link
International Federation of Gynecology and Obstetrics	Caloric intake should be calculated based on pre-pregnancy BMI and desirable weight gain; Caloric intake may be reduced by 30%, but not below 1600−1800 kcal/d; for women with diabetic nephropathy, protein may be lowered to 0.6−0.8 g/kg ideal body weight.	Carbohydrate intake should be limited to 35%–45% of total calories, with a minimum of 175 g CHO per day, distributed in three small-to-moderate sized meals and 2−4 snacks.	*M. Hod* et al./*International Journal of Gynecology and Obstetrics 131 S3 (2015) S173–S211*
Endocrine Society	Medical nutrition therapy is recommended for all pregnant women with overt or gestational diabetes to help achieve and maintain desired glycemic control while providing essential nutrient requirements.	Carbohydrate should be limited to 35% to 45% of total calories, distributed in 3 small-to-moderate-sized meals and 2 to 4 snacks including an evening snack	Blumer I., Hadar E., Haddan DR., et al., Diabetes and Pregnancy: An Endocrine Society Clinical Practice Guideline. J Clin Endo Metab 2013:98:4227–4249.
American College of Obstetrics and Gynecologists	Eat regular meals throughout the day; three meals and two–three snacks per day. Gain healthy amount of weight.	Complex CHO are recommended over simple CHO because they are digested more slowly, are less likely to produce significant postprandial hyperglycemia, and potentially reduce insulin resistance.	Obstetrics & Gynecology. 131(2):e49–e64, FEBRUARY 2018 OI: 10.1097/AOG.0000000000002501 PMID: 29370047 Issn Print: 0029–7844 Publication Date: February 2018
National Institute for Health and Care Excellence (NICE) guidelines	Advise women to eat a healthy diet during pregnancy, refer all women with gestational diabetes to a dietitian.	Foods with a low glycemic index should replace those with a high glycemic index.	NICE National Institute for Health and Care Excellence Guideline. Diabetes in pregnancy: Management from preconception to the postnatal period. Published: 25 February 2015 www.nice.org.uk/guidance/ng3
Diabetes Canada	Meal planning for women with GDM should emphasize a healthy diet during pregnancy.	Women should consume a minimum of 175 g/day of CHO, distributed over 3 moderate-sized meals and 2 or more snacks (1 of which should be at bedtime), replacing high-GI foods with low-GI ones.	*Feig DS, Berger H., Donovan L., et al., Diabetes and Pregnancy. Diabetes Canada 2018. Clinical Practice Guidelines for the Prevention and Management of Diabetes in Canada: Pharmacologic Glycemic Management of Type 2 Diabetes in Adults. Can J Diabetes 2018;42(Suppl 1):S255–S282.*
American Academy of Nutrition and Dietetics	A registered dietitian nutritionist (or international equivalent) should provide Medical Nutrition Therapy that includes an individual nutrition prescription and nutrition counseling for all women diagnosed with GDM.	All pregnant women should eat a minimum of 157 g CHO and 28 g fiber. The amount and type of CHO should be individualized based on nutrition assessment, treatment goals, blood glucose response and patient needs. Three meals and 2 or more snacks helps to distribute CHO intake and reduce postprandial blood glucose elevations.	Duarte Gardea et al., Academy of Nutrition and Dietetics Gestational Diabetes Evidence-Based Nutrition Practice Guideline Journal of the Academy of Nutrition and Dietetics. September 2018 Volume 118, Issue 9, Pages 1719–1742. https://doi.org/10.1016/j.jand.2018.03.014
American Diabetes Association	The food plan should be based on a nutrition assessment with guidance from the Dietary Reference Intakes.	All pregnant women should eat a minimum of 175 g total CHO and 28 g fiber. For women with GDM, the amount and type of CHO will impact glucose levels, especially post-meal excursions.	American Diabetes Association. 14. Management of Diabetes in Pregnancy: Standards of Medical Care in Diabetes. 2019 Diabetes Care 2019;42(Suppl. 1): S165–S172|https://doi.org/10.2337/dc19-S014
